# Circulating Nucleosomes Respond to Moderate Aerobic Exercise: A Pilot Study in Healthy Individuals and Cancer Patients

**DOI:** 10.3390/diagnostics16213633

**Published:** 2026-11-03

**Authors:** Elisa Danese, Cantor Tarperi, Mauro Di Ruvo, Marco Tosi, Laura Pighi, Matteo Gelati, Alice Avancini, Lorenzo Budel, Sara Pilotto, Roberta Ferraro, Gian Luca Salvagno, Federico Schena, Giuseppe Lippi

**Affiliations:** 1 Clinical Biochemistry Section, Department of Engineering for Innovation Medicine, University of Verona, 37134 Verona, Italy; elisa.danese@univr.it (E.D.); mauro.diruvo@studenti.univr.it (M.D.R.); marco.tosi@studenti.univr.it (M.T.); laura.pighi2@aovr.veneto.it (L.P.); matteo.gelati@univr.it (M.G.); roberta.ferraro@studenti.univr.it (R.F.); gianluca.salvagno@univr.it (G.L.S.); 2 Department of Neuroscience, Biomedicine and Movement Sciences, University of Verona, 37134 Verona, Italy; cantor.tarperi@univr.it (C.T.); alice.avancini@univr.it (A.A.); lorenzo.budel@univr.it (L.B.); federico.schena@univr.it (F.S.); 3 Section of Innovation Biomedicine Oncology, Department of Engineering for Innovation Medicine (DIMI), University of Verona and University and Hospital Trust (AOUI) of Verona, 37134 Verona, Italy; sara.pilotto@univr.it

**Keywords:** circulating nucleosomes, H3.1 histone, cancer, exercise biomarkers

## Abstract

**Background/Objectives**: Circulating nucleosomes containing H3.1 represent a distinct fraction of extracellular chromatin. Although strenuous exercise is known to increase circulating extracellular chromatin components, it remains unclear whether H3.1-containing nucleosomes also respond to moderate physiological stress and whether this response varies with the underlying biological condition. **Methods**: Sixteen healthy subjects and 13 subjects with cancer completed a standardized 5 km walking exercise. Venous blood samples were collected immediately before and after exercise, and circulating H3.1-containing nucleosomes were quantified in K2-EDTA plasma with an automated immunoassay. Within-group and between-group comparisons were performed using non-parametric tests. Relative exercise-induced changes and age-adjusted analyses were evaluated as sensitivity analyses. **Results**: Circulating H3.1-containing nucleosome concentrations increased significantly after exercise in both healthy subjects (*p* < 0.0001) and those with cancer (*p* = 0.001). Cancer patients displayed higher baseline concentrations (*p* = 0.0056), whereas post-exercise concentrations were comparable between groups (*p* = 0.840). The absolute exercise-induced increase was significantly higher in healthy subjects (*p* = 0.028). This difference was confirmed when the response was expressed relative to individual baseline concentrations (median increase: 124.7% [IQR 63.9–191.5%] vs. 36.5% [20.6–42.0%]; *p* = 0.0009) and persisted after age adjustment (*p* = 0.0046). **Conclusions**: Circulating H3.1-containing nucleosomes respond to moderate aerobic exercise, with distinct patterns in healthy subjects and those with cancer. These preliminary findings support further investigation of H3.1-containing nucleosomal chromatin as a dynamic marker of the biological response to exercise.

## 1. Introduction

Circulating nucleosomes are cell-free chromatin complexes composed of DNA wrapped around a core of histone proteins [1]. H3.1 is a canonical replication-dependent histone H3 isoform incorporated into nucleosomal chromatin. Histone variants differ in their patterns of expression and chromatin incorporation and contribute to the structural and functional heterogeneity of nucleosomes [2]. The quantification of circulating H3.1-containing nucleosomes therefore detects extracellular nucleosomal chromatin containing this histone variant, rather than free H3.1 or cell-free DNA (cfDNA) alone [3]. This molecular specificity is analytically significant because extracellular chromatin may circulate in different molecular forms not necessarily captured by the same analytical approaches. Nucleosomes can enter the circulation as a consequence of cellular turnover and cell death, as well as through regulated extracellular chromatin release. Their circulating concentrations reflect the balance between release and clearance and may provide information on ongoing changes in tissue homeostasis [4,5,6]. Accordingly, the measurement of intact nucleosomes provides information that is related to, but not interchangeable with, that obtained from assays targeting cfDNA or individual extracellular histones.

Circulating nucleosomes have been investigated extensively in oncology. Increased concentrations were reported in several solid and hematological malignancies and have been associated with tumor-related cellular turnover, cell death, and treatment-associated changes [7,8,9,10]. More recent evidence has further highlighted the potential relevance of circulating nucleosomes as biomarkers for cancer detection and treatment monitoring [11]. However, circulating nucleosomes are not tumor-specific, since their release may also occur under physiological conditions and in non-malignant pathological processes. Therefore, their concentrations should be interpreted in relation to the underlying biological and clinical context rather than as direct indicators of tumor burden. Rather than reflecting a single disease-specific process, circulating nucleosome concentrations may integrate changes in cellular turnover, extracellular chromatin release, and clearance occurring in different biological settings. This makes cancer patients an especially important population for investigating whether an acute physiological stimulus elicits a nucleosome response different from that observed in healthy individuals.

Exercise is a controlled physiological stimulus that can induce rapid changes in circulating chromatin-derived molecules. Most evidence derives from studies of cell-free DNA (cfDNA), which increases rapidly following acute exercise and originates predominantly from hematopoietic cells, especially mature neutrophils [12,13,14]. Recent investigation using cell-type-specific molecular approaches have identified contributions from several innate immune-cell populations, including neutrophils, dendritic cells, and macrophages, to exercise-induced cfDNA. Their relative contributions appear to depend on exercise intensity and training status [15]. Altogether, these observations indicate that exercise-induced extracellular chromatin release is a complex biological response involving immune-cell activation and cannot be attributed exclusively to tissue injury or irreversible cell death. Nevertheless, cfDNA measurements do not distinguish DNA that circulates in nucleosomal complexes from other extracellular DNA forms, so that findings from cfDNA assays cannot be directly extrapolated to intact circulating nucleosomes. This analytical distinction is especially important in exercise studies because extracellular chromatin components may vary in their cellular origin, mechanisms of release, circulating stability, and clearance kinetics.

Evidence on exercise-induced changes in circulating histones and nucleosomes is considerably more limited. Repeated exhaustive exercise has been associated with increased concentrations of circulating and citrullinated histone H3 [16]. Histone citrullination is involved in chromatin decondensation during neutrophil extracellular trap formation, providing a potential link between exercise-induced immune-cell activation and extracellular chromatin release. Measurements of circulating histones, namely post-translationally modified forms such as citrullinated H3, may hence provide information on specific pathways of chromatin release and neutrophil activation.

However, circulating histones are analytically and biologically distinct from intact nucleosomes. Accordingly, changes in circulating histones may not necessarily parallel those of intact nucleosomes, whilst measurement of intact nucleosomes retains information on the nucleosomal organization of extracellular chromatin, thus capturing a different aspect of the biological response to exercise. More direct evidence comes from recreational marathon runners, in whom circulating nucleosome concentrations increased immediately after the race and subsequently declined during recovery [17]. Therefore, available evidence indicates that strenuous endurance exercise can transiently modify circulating nucleosome concentrations, but whether a shorter and substantially less demanding physiological stimulus is sufficient to elicit a measurable response remains unknown. Moreover, the response of circulating nucleosomes specifically containing the H3.1 histone variant to moderate aerobic exercise has not been characterized.

This question is particularly relevant in subjects with cancer, in whom circulating nucleosome concentrations may already be elevated due to increased cellular turnover. Cancer and its treatment may also affect immune-cell function, cell death, and extracellular chromatin processing, potentially modifying both baseline nucleosome concentrations and their response to an acute physiological stimulus. Exercise is increasingly incorporated into cancer care [18], yet the acute circulating nucleosome response to moderate exercise has not been compared between healthy subjects and those with cancer. Determining whether the same standardized exercise challenge produces different H3.1-containing nucleosome dynamics in these two populations may provide valuable insights into how the underlying biological condition influences the acute extracellular chromatin response.

Therefore, the present study was aimed to investigate whether a standardized 5 km walking exercise elicits measurable changes in circulating H3.1-containing nucleosomes, and whether the magnitude of this response differs between healthy subjects and those with cancer.

## 2. Materials and Methods

### 2.1. Study Design

Participants were enrolled during the 2025 edition of the Run for Science project, a multidisciplinary research initiative coordinated by the University of Verona aimed at investigating the physiological and molecular responses to exercise [19]. The 2025 edition focused specifically on the effects of exercise in individuals with cancer and was held in Verona (Italy) on 12 April 2025. The demographic, clinical, and baseline laboratory characteristics of the study population are summarized in Table 1. The cancer group included subjects with a heterogeneous range of solid malignancies, including lung, breast, colorectal, and head and neck cancers, across stages I–IV and undergoing active anticancer treatment (Supplementary Table S1).

All participants completed a standardized 5 km walking exercise performed outdoors on a flat urban course. Venous blood samples were collected immediately before the start of the walk and immediately after its completion, in accordance with the standardized Run for Science protocol. Weather conditions during testing were stable, with sunny skies, ambient temperatures ranging between 16 and 19 °C, and light wind (5–6 km/h). The protocol of the Run for Science project was approved by the Ethics Committee of the Department of Neuroscience, Biomedicine and Movement Sciences of the University of Verona (protocol no. 165038, dated 28 June 2016). All participants provided written informed consent before enrolment in the study.

### 2.2. Measurement of Circulating H3.1-Containing Nucleosomes

Circulating H3.1-containing nucleosomes were quantified in K2-EDTA plasma using the Nu.Q^®^ H3.1 immunoassay (Belgian Volition SRL, Isnes, Belgium) on the IDS-isys automated immunoanalyzer (Immunodiagnostic Systems Ltd., Boldon Colliery, UK), according to manufacturer’s instructions. The assay is based on a sandwich immunoassay that employs an antibody directed against histone H3.1, together with a nucleosome-specific detection antibody, thereby selectively quantifying intact circulating H3.1-containing nucleosomes [20]. All samples were analyzed on the same analytical platform using the same reagent lot and under identical operating conditions. Results were reported as circulating concentrations of H3.1-containing nucleosomes (ng/mL).

### 2.3. Statistical Analysis

Statistical analyses were performed using GraphPad Prism version 11 (GraphPad Software, Boston, MA, USA). Data distribution was assessed using the Shapiro–Wilk test. Given the small sample size and non-normal distributions of some variables, continuous data are presented as medians and interquartile ranges (IQRs), and primary unadjusted comparisons were performed using non-parametric tests. Within-group changes in circulating H3.1-containing nucleosome concentrations were assessed using the two-tailed Wilcoxon matched-pairs signed-rank test, and between-group comparisons using the two-tailed Mann–Whitney U test. The absolute exercise-induced change was calculated as the post-exercise concentration minus the baseline concentration. As a sensitivity analysis, the relative exercise-induced change was calculated as [(post-exercise − baseline)/baseline] × 100, and compared between groups using the Mann–Whitney U test. Linear regression models, including study group and age as independent variables, were additionally used to assess between-group differences in baseline concentrations and absolute exercise-induced changes after adjustment for age. Changes in selected hematological and biochemical parameters (Supplementary Table S2) were assessed using the Wilcoxon matched-pairs signed-rank test; between-group differences in hematological and biochemical parameters were assessed using the Mann–Whitney U test. Associations between the absolute exercise-induced change in H3.1-containing nucleosomes and concomitant changes in hematological and biochemical parameters were assessed using Spearman’s rank correlation coefficient. These analyses were exploratory, with no adjustment for multiple comparisons.

## 3. Results

All participants completed the 5 km walking exercise, with a mean completion time of 73′10″ in healthy subjects (mean speed: 4.13 ± 0.34 km/h) and 77′43″ in those with cancer (mean speed: 3.91 ± 0.47 km/h; *p* = 0.115). Circulating H3.1-containing nucleosome concentrations and exercise-induced changes are summarized in Table 2. At baseline, subjects with cancer displayed significantly higher concentrations than healthy subjects (*p* = 0.0056), whereas post-exercise concentrations were comparable between groups (*p* = 0.840).

**Figure 1:**
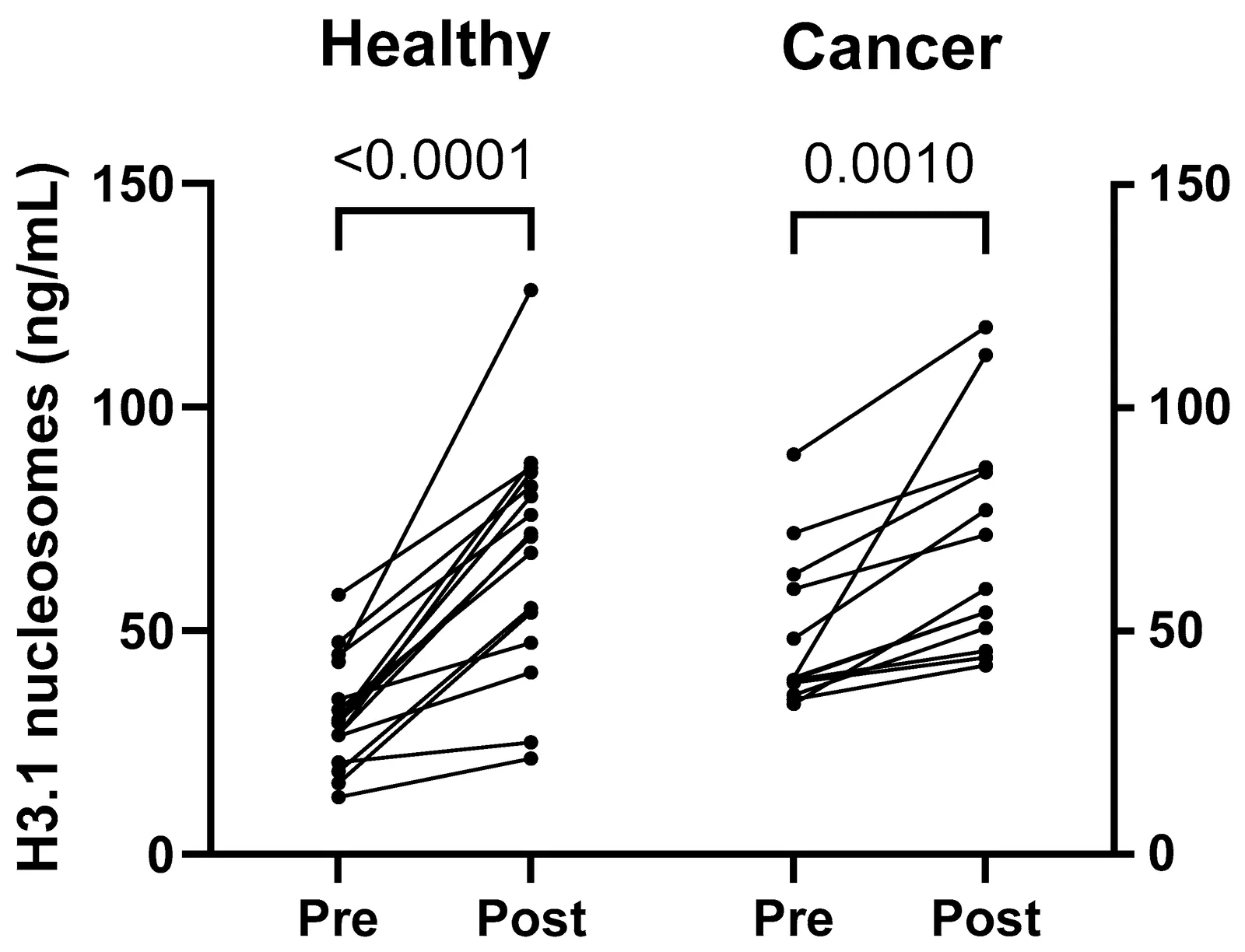
Individual responses of circulating H3.1-containing nucleosomes to the 5 km walking exercise.

Circulating H3.1-containing nucleosome concentrations increased significantly after the 5 km walking exercise in both healthy subjects (*p* < 0.0001) and those with cancer (*p* = 0.001). Individual paired pre- and post-exercise concentrations are shown in Figure 1.

**Figure 2:**
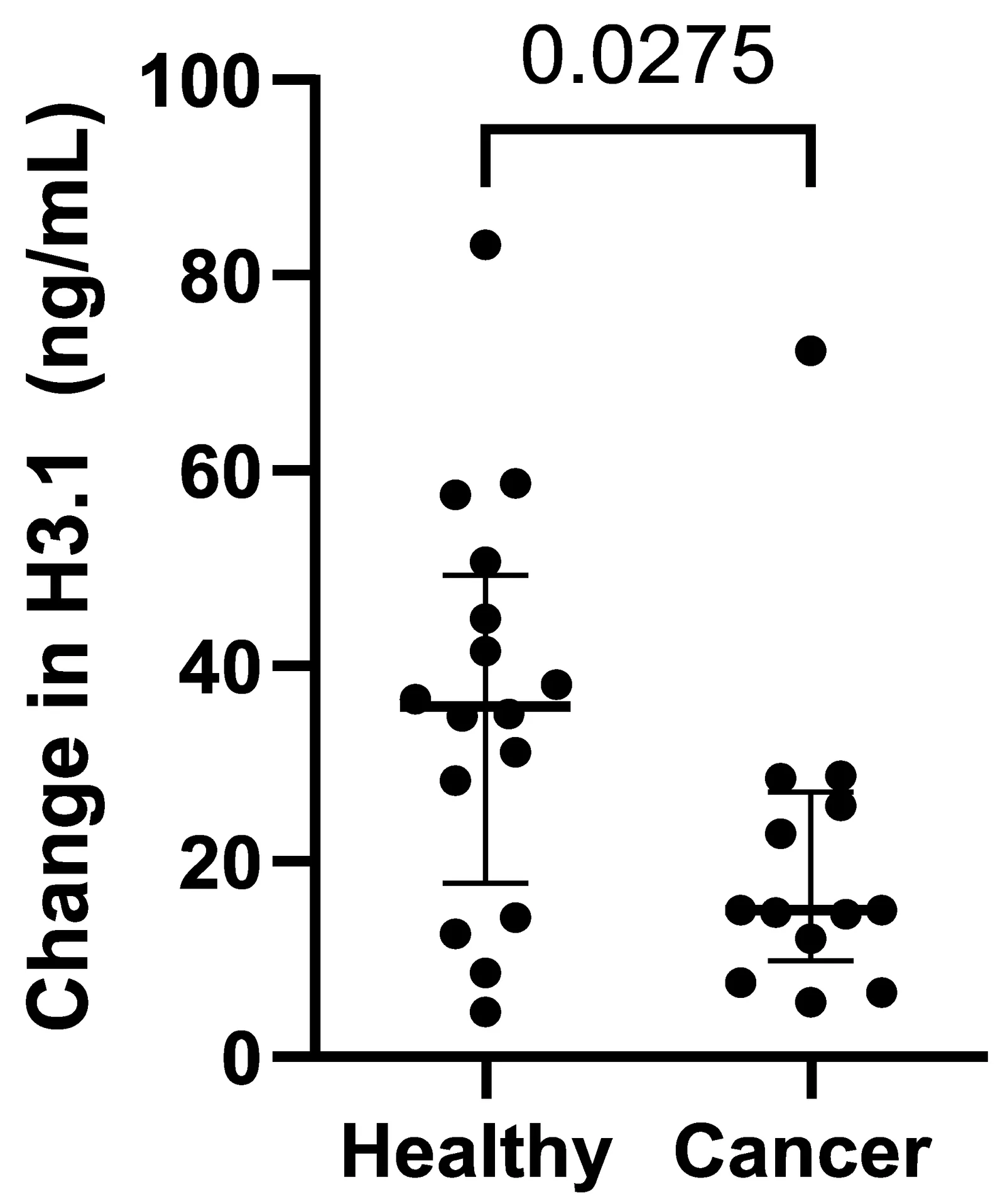
Absolute exercise-induced change in circulating H3.1-containing nucleosomes.

The absolute exercise-induced change was significantly higher in healthy subjects than in subjects with cancer (*p* = 0.028; Figure 2). This difference was also observed when the response was expressed relative to individual baseline concentrations (median relative change, 124.7% [IQR 63.9–191.5%] vs. 36.5% [20.6–42.0%], respectively; *p* = 0.0009). After age adjustment, both the higher baseline concentrations in subjects with cancer (*p* = 0.040) and their smaller absolute exercise-induced change (*p* = 0.0046) remained significant.

Exploratory hematological and biochemical analyses are reported in Supplementary Table S2. The neutrophil-to-lymphocyte ratio (NLR) increased significantly following exercise in both groups, whereas the magnitude of the exercise-induced change did not differ between healthy subjects and subjects with cancer (*p* = 0.983). Exercise-induced changes in H3.1-containing nucleosomes were not significantly associated with concomitant changes in blood cell counts, NLR, or creatine kinase.

Changes in hematocrit were not associated with exercise-induced changes in H3.1-containing nucleosomes (ρ = −0.012, *p* = 0.953) and did not differ between groups (*p* = 0.862), arguing against hemoconcentration as a major determinant of the observed nucleosome response.

Circulating H3.1-containing nucleosome concentrations measured before (Pre) and immediately after (Post) the standardized 5 km walking exercise in healthy subjects (A) and subjects with cancer (B). Each line represents paired measurements obtained from the same participant.

Exercise-induced changes in circulating H3.1-containing nucleosome concentrations, calculated as post-exercise minus baseline values, following the standardized 5 km walking exercise in healthy subjects and subjects with cancer. Individual observations are shown together with the median and interquartile range.

## 4. Discussion

The present study shows that circulating H3.1-containing nucleosomes are responsive to moderate aerobic exercise. A standardized 5 km walking exercise significantly increased their circulating concentrations in both healthy individuals and subjects with cancer. Despite higher baseline concentrations, subjects with cancer showed a smaller exercise-induced response, resulting in comparable post-exercise concentrations between groups. Sensitivity analyses supported the robustness of this between-group difference.

Our findings extend previous observations on exercise-induced changes in circulating chromatin components. Increased circulating histone H3 has been reported following repeated exhaustive exercise [16]. Circulating nucleosome concentrations have also been shown to increase after marathon running [17]. Altogether, these studies indicate that strenuous exercise can affect various circulating chromatin components. The present study extends this limited evidence by focusing specifically on intact circulating nucleosomes containing the H3.1 histone variant under a substantially less demanding exercise condition.

The attenuated exercise-induced response observed in subjects with cancer is particularly noteworthy. Their higher baseline nucleosome concentrations are consistent with previous observations in solid and hematological malignancies [7,8,9]. Because subjects with cancer were older than healthy subjects, age may have contributed to the baseline difference; nevertheless, the higher baseline concentrations in subjects with cancer remained significant after age adjustment. Importantly, the smaller exercise-induced response also persisted after adjustment for age and was further supported when expressed relative to individual baseline concentrations, supporting the robustness of this finding.

The combination of higher baseline H3.1-containing nucleosome concentrations, a smaller exercise-induced increase, and comparable post-exercise concentrations suggest that the observed pattern may reflect differences in the dynamic balance between extracellular chromatin release and clearance. Acute exercise has been shown to promote neutrophil extracellular chromatin release together with extracellular DNA degradation [21]. Circulating H3.1-containing nucleosomes have also recently been associated with systemic inflammatory states, supporting their potential responsiveness to biological processes beyond tumor-related cell turnover [3]. In patients with different types of cancer, disease- or treatment-related alterations in cellular turnover, immune function, inflammation, or chromatin processing could potentially modify these dynamics. Therefore, the attenuated response may reflect differences in stimulus-induced chromatin release, clearance, or both, although these mechanisms remain hypothetical and cannot be distinguished by the present study.

Exploratory hematological analyses provide additional context to the observed response. NLR, a widely used marker of systemic inflammatory response in cancer [22], increased following exercise in both groups (Supplementary Table S2), without a difference in the magnitude of change between groups. Moreover, exercise-induced changes in H3.1-containing nucleosomes were not associated with changes in leukocyte or neutrophil counts or NLR. Thus, quantitative leukocyte mobilization alone does not appear to account for the distinct H3.1-containing nucleosome response, although a contribution from immune-cell activation cannot be excluded. This is a meaningful distinction because changes in circulating cell numbers do not necessarily reflect functional activation or extracellular chromatin release by these cells. Accordingly, dedicated studies combining nucleosome measurements with markers of neutrophil activation, extracellular trap formation, and nuclease activity will be required to clarify the cellular mechanisms underlying the observed response.

A methodological strength of the present study is the automated quantification of intact circulating H3.1-containing nucleosomes using a standardized immunoassay. This approach differs conceptually and analytically from measuring cfDNA or free extracellular histones, as these analytes represent distinct forms of extracellular chromatin and may provide complementary biological information. cfDNA assays primarily quantify extracellular DNA without specifically requiring preservation of its nucleosomal organization, whereas assays targeting free extracellular histones detect histone proteins independently of their incorporation into intact nucleosomes. By contrast, the present assay requires recognition of nucleosomal chromatin containing the H3.1 histone variant and specifically interrogates this structurally defined component of extracellular chromatin [20]. These differences may also have biological relevance, because the release, persistence, and clearance of cfDNA, free histones, and intact nucleosomes may not occur in parallel. Each analytical approach has potential advantages: cfDNA provides a sensitive measure of extracellular DNA release and has been extensively characterized in exercise physiology [12,13,14]; extracellular histones may provide information on histone release and specific post-translational modifications [16]; whereas intact nucleosome measurements retain information on the nucleosomal organization of circulating chromatin [17]. The latter may provide a complementary perspective on exercise-induced extracellular chromatin dynamics rather than serving as an alternative measure of the same biological signal [23].

Our findings may also have some potential clinical implications. Exercise is increasingly incorporated into supportive cancer care, yet circulating biomarkers that capture the acute biological response to a standardized exercise stimulus remain limited. The observation that H3.1-containing nucleosomes increased following a moderate walking exercise in both healthy subjects and subjects with cancer suggests that this circulating chromatin component is sufficiently dynamic to detect relatively modest physiological perturbations. Of particular interest, cancer patients displayed an attenuated response despite higher baseline concentrations, suggesting that the exercise-induced change may provide information distinct from a single resting measurement. This dynamic behavior may be relevant when considering biomarkers of physiological response, for which the magnitude of change following a standardized challenge may convey information not apparent from baseline concentrations alone. If confirmed in larger and clinically homogeneous populations, monitoring H3.1-containing nucleosome dynamics could contribute to the biological characterization of individual responses to exercise and potentially complement conventional physiological measures in exercise-oncology research. However, the present findings should be considered hypothesis-generating, and further studies are required to determine whether these changes are associated with exercise tolerance, training adaptation, treatment status, or clinically relevant outcomes.

The main limitations of the present study are the relatively small sample size and the clinical heterogeneity of the cancer group, which limit statistical power and preclude comprehensive adjustment for potential confounding factors or assessment of the influence of tumor type, disease status, and anticancer treatment. Although sensitivity analyses accounting for age supported the robustness of the main findings, residual confounding related to other individual characteristics cannot be excluded. Nevertheless, recruiting clinically eligible subjects with cancer who are willing to participate in exercise-based experimental studies is challenging, and the present study should therefore be regarded as a pilot investigation providing preliminary evidence of the response of circulating H3.1-containing nucleosomes to moderate aerobic exercise in this population. In addition, sampling immediately before and after exercise prevented characterization of H3.1-containing nucleosome kinetics during recovery. Finally, the lack of specific markers of immune-cell activation, extracellular trap formation, and nuclease activity limits further mechanistic interpretation of the observed response.

## 5. Conclusions

In conclusion, a standardized 5 km walking exercise induces an acute increase in circulating H3.1-containing nucleosomes in both healthy subjects and cancer patients. The higher baseline concentrations observed in subjects with cancer could, at least in part, contribute to the smaller absolute increase observed after exercise. However, the difference between groups remained evident when the exercise-induced response was expressed relative to the individual baseline concentration and after age adjustment, suggesting that the attenuated response cannot be only explained by baseline concentrations or age differences. This finding may indicate differences in the balance between exercise-induced chromatin release and subsequent clearance in subjects with cancer, although the mechanisms underlying this observation remain uncertain. These findings broaden current evidence on exercise-induced extracellular chromatin dynamics and provide a basis for further investigation of the mechanisms regulating H3.1-containing nucleosomes during physical activity.

## Figures and Tables

**Table 1:** Demographic, clinical, and baseline laboratory characteristics of the study population.

Demographic and Clinical Characteristics	Healthy Subjects (*n* = 16)	Cancer Patients (*n* = 13)	*p*-Value
Age, years	50 (35–61)	63 (53–69)	0.025
Sex, female/male	7/9	9/4	0.264
**Baseline laboratory parameters**			
Red blood cells, ×10^12^/L	5.04 (4.75–5.29)	4.27 (3.75–4.45)	0.002
Hemoglobin, g/L	143.5 (131.0–154.8)	128.0 (122.0–143.0)	0.028
Platelets, ×10^9^/L	243.5 (225.5–301.5)	222.0 (167.0–253.0)	0.109
White blood cells, ×10^9^/L	6.10 (5.28–6.64)	5.72 (4.57–5.92)	0.211
Neutrophils, ×10^9^/L	3.15 (2.80–3.71)	2.53 (2.32–3.31)	0.100
Lymphocytes, ×10^9^/L	2.02 (1.48–2.33)	1.83 (1.29–2.31)	0.569
Monocytes, ×10^9^/L	0.54 (0.45–0.67)	0.45 (0.42–0.55)	0.323
Eosinophils, ×10^9^/L	0.14 (0.12–0.19)	0.10 (0.07–0.20)	0.263
Neutrophil-to-lymphocyte ratio (NLR)	1.60 (1.45–1.95)	1.77 (1.06–2.16)	0.742
Creatine kinase (CK), U/L	125 (96–170)	119 (80–147)	0.469

Data are presented as median (interquartile range) or number, as appropriate.

**Table 2:** Circulating H3.1-containing nucleosome concentrations before and after the 5 km walking exercise.

Variable	Healthy Subjects (*n* = 16)	Cancer Patients (*n* = 13)	*p* Value
Baseline (ng/mL)	29.5 (26.5–39.0)	39.5 (37.1–60.9)	0.0056
Post-exercise (ng/mL)	73.9 (54.6–84.9)	59.4 (49.0–81.4)	0.840
Absolute exercise-induced change (ng/mL)	35.2 (22.6–47.0)	15.0 (12.1–25.7)	0.028

Data are presented as median (interquartile range).

## Data Availability

Data are available on request to the authors.

## Supplementary Materials

The following supporting information can be downloaded at: https://www.mdpi.com/article/10.3390/diagnostics16213633/s1. Table S1: Cancer group clinical and treatment characteristics; Table S2: Hematological and biochemical responses to the 5 km walking exercise.

## Abbreviations

The following abbreviations are used in this manuscript:

NLR	Neutrophil-to-lymphocyte ratio
CK	Creatinine Kinase
IQRs	Interquartile ranges
PRE	Pre-exercise
POST	Post-exercise
