# Supplementary material for: Circulating Nucleosomes Respond to Moderate Aerobic Exercise: A Pilot Study in Healthy Individuals and Cancer Patients
Source: Diagnostics (Basel). 2026 Nov 3;16(21):3633. doi: 10.3390/diagnostics16213633 (PMC13763562; doi:10.3390/diagnostics16213633)
Supplement: Supplementary file 1 [file diagnostics-16-03633-s001.zip › diagnostics-4572840-supplementary.pdf]

Supplementary Table S1 Cancer group clinical and treatment characteristics.

		n	%
**Total subjects**		13	100
**Cancer site**	Breast	7	53.8
	Prostate	1	7.7
	Lung	2	15.4
	Gastrointestinal	2	15.4
	Head & Neck	1	7.7
**Cancer stage**	I	3	23.1
	II	2	15.4
	III	4	30.8
	IV	4	30.8
**Type of treatment**	Chemotherapy	5	38.5
	Radiotherapy	3	23.1
	Immunotherapy	1	7.7
	Target therapy	2	15.4
	Hormone therapy	7	53.8
**Timing of treatment**	Ongoing	11	84.6
	Completed	2	15.4

Supplementary Table S2 Hematological and biochemical responses to the 5-km walking exercise

Parameter	Healthy PRE	Healthy POST	P value	Subjects with cancer PRE	Subjects with cancer POST	P value
Hematocrit, L/L	0.45 (0.42–0.47)	0.44 (0.40–0.46)	0.012	0.41 (0.38–0.43)	0.38 (0.36–0.43)	0.034
Erythrocytes, $\times 10^{12}$/L	5.04 (4.75–5.29)	4.98 (4.66–5.12)	0.006	4.27 (3.75–4.45)	4.11 (3.69–4.55)	0.057
Hemoglobin, g/L	143.5 (131.0–154.8)	141.5 (131.5–156.0)	0.069	128.0 (122.0–143.0)	125.0 (115.0–141.0)	0.064
Platelets, $\times 10^{9}$/L	243.5 (225.5–301.5)	253.0 (238.5–299.5)	0.003	222.0 (167.0–253.0)	224.0 (180.0–287.0)	0.147
Leukocytes, $\times 10^{9}$/L	6.10 (5.28–6.64)	6.20 (5.56–7.06)	0.039	5.72 (4.57–5.92)	5.79 (4.53–6.48)	0.162
Neutrophils, $\times 10^{9}$/L	3.15 (2.80–3.71)	3.59 (3.27–4.38)	0.0004	2.53 (2.32–3.31)	2.72 (2.50–3.58)	0.016
Lymphocytes, $\times 10^{9}$/L	2.02 (1.48–2.33)	1.71 (1.54–2.15)	0.278	1.83 (1.29–2.31)	1.75 (1.21–2.07)	0.311
Monocytes, $\times 10^{9}$/L	0.54 (0.45–0.67)	0.52 (0.41–0.65)	0.501	0.45 (0.42–0.55)	0.46 (0.34–0.53)	0.839
Eosinophils, $\times 10^{9}$/L	0.14 (0.12–0.19)	0.09 (0.05–0.12)	0.0007	0.10 (0.07–0.20)	0.06 (0.04–0.13)	0.0007
NLR	1.60 (1.45–1.95)	2.09 (1.58–2.74)	0.034	1.77 (1.06–2.16)	1.94 (1.45–2.37)	0.017
CK, U/L	125 (96–170)	140 (112–182)	0.0004	119 (80–147)	133 (90–168)	0.0005

Data are presented as median (interquartile range). PRE, pre-exercise; POST, post-exercise; CK, creatine kinase. NLR: neutrophil-to-lymphocyte ratio
